# Wearable Biosensing to Predict Imminent Aggressive Behavior in Psychiatric Inpatient Youths With Autism

**DOI:** 10.1001/jamanetworkopen.2023.48898

**Published:** 2023-12-21

**Authors:** Tales Imbiriba, Ahmet Demirkaya, Ashutosh Singh, Deniz Erdogmus, Matthew S. Goodwin

**Affiliations:** 1 College of Engineering, Northeastern University, Boston, Massachusetts; 2 Bouvé College of Health Sciences, Northeastern University, Boston, Massachusetts; 3 Khoury College of Computer Sciences, Northeastern University, Boston, Massachusetts

## Abstract

**Importance:**

Aggressive behavior is a prevalent and challenging issue in individuals with autism.

**Objective:**

To investigate whether changes in peripheral physiology recorded by a wearable biosensor and machine learning can be used to predict imminent aggressive behavior before it occurs in inpatient youths with autism.

**Design, Setting, and Participants:**

This noninterventional prognostic study used data collected from March 2019 to March 2020 from 4 primary care psychiatric inpatient hospitals. Enrolled participants were 86 psychiatric inpatients with confirmed diagnoses of autism exhibiting operationally defined self-injurious behavior, emotion dysregulation, or aggression toward others; 16 individuals were not included (18.6%) because they would not wear the biosensor (8 individuals) or were discharged before an observation could be made (8 individuals). Data were analyzed from March 2020 through October 2023.

**Main Outcomes and Measures:**

Research staff performed live behavioral coding of aggressive behavior while inpatient study participants wore a commercially available biosensor that recorded peripheral physiological signals (cardiovascular activity, electrodermal activity, and motion). Logistic regression, support vector machines, neural networks, and domain adaptation were used to analyze time-series features extracted from biosensor data. Area under the receiver operating characteristic curve (AUROC) values were used to evaluate the performance of population- and person-dependent models.

**Results:**

There were 70 study participants (mean [range; SD] age, 11.9 [5-19; 3.5] years; 62 males [88.6%]; 1 Asian [1.4%], 5 Black [7.1%], 1 Native Hawaiian or Other Pacific Islander [1.4%], and 63 White [90.0%]; 5 Hispanic [7.5%] and 62 non-Hispanic [92.5%] among 67 individuals with ethnicity data). Nearly half of the population (32 individuals [45.7%]) was minimally verbal, and 30 individuals (42.8%) had an intellectual disability. Participant length of inpatient hospital stay ranged from 8 to 201 days, and the mean (SD) length was 37.28 (33.95) days. A total of 429 naturalistic observational coding sessions were recorded, totaling 497 hours, wherein 6665 aggressive behaviors were documented, including self-injury (3983 behaviors [59.8%]), emotion dysregulation (2063 behaviors [31.0%]), and aggression toward others (619 behaviors [9.3%]). Logistic regression was the best-performing overall classifier across all experiments; for example, it predicted aggressive behavior 3 minutes before onset with a mean AUROC of 0.80 (95% CI, 0.79-0.81).

**Conclusions and Relevance:**

This study replicated and extended previous findings suggesting that machine learning analyses of preceding changes in peripheral physiology may be used to predict imminent aggressive behaviors before they occur in inpatient youths with autism. Further research will explore clinical implications and the potential for personalized interventions.

## Key Points

**Question:**

Can a wearable biosensor and machine learning be used to predict imminent aggression before it occurs in youths with autism who are psychiatric inpatients?

**Findings:**

In this prognostic study involving 70 youths with autism across 4 psychiatric inpatient hospitals, the best-performing overall classifier was logistic regression, which predicted aggressive behavior 3 minutes before it occurred with a mean area under the receiver operating characteristic curve of 0.80.

**Meaning:**

This finding suggests that wearable biosensing and machine learning may hold promise for identifying objective indicators of impending aggressive behaviors in youths with autism who are psychiatric inpatients.

## Teaser

This prognostic study investigates the use of wearable biosensors and machine learning for predicting aggressive behaviors in youths with autism who are psychiatric inpatients.

## Introduction

Autism is one of the most common childhood disorders (occurring in 1 in 36 children).^1^ Aggressive behavior (including self-injury, tantrums, meltdowns, property destruction, and aggression toward others [ATO]) occurs in as much as 80% of children and adolescents with autism,^2,3,4^ ranks among the most common causes for referral to behavioral health care services,^5^ and incurs high health care costs.^6,7,8,9,10^ Several factors make it difficult for youths with autism to regulate their emotions and self-report their internal states^11^; 30% to 40% are minimally verbal,^12^ and those who are fluently verbal often have poor emotional insight and self-awareness.^13^ This predicament can make aggressive behavior unpredictable and thus dangerous, creating a barrier to accessing the community, therapy services, clinicians, and educational placements. Families report that aggressive behavior increases their stress, isolation, and financial burden and decreases available support options because they fear putting their child with autism into environments that may result in unexpected aggressive behavior.^14,15^ Aggressive behavior also affects support professionals, leading to compensatory payments for injury, increased numbers of sick days, and higher turnover rates.^16,17^ This challenging situation can demoralize parents and clinicians, accelerate negative patient trajectories, and lead to homebound or residential living placement care, collectively decreasing quality of life while increasing costs.

Peripheral physiology is a promising objective indicator of aggressive behavior.^18,19^ While significant heterogeneity in individuals with autism exists, atypical autonomic reactivity is a common feature^20,21,22^ and can putatively occasion maladaptive behavior when demands exceed an individual’s coping ability.^23,24^

In prior work,^25^ we recorded peripheral physiological (cardiovascular and electrodermal activity) and motion (accelerometry) signals from a wearable biosensor worn by 20 youths with autism (ages 6-17 years; 75% male; 85% minimally verbal) during naturalistic observation sessions with concurrent behavioral coding in a single specialized inpatient psychiatry unit. Our objective was to test the hypothesis that we could use preceding physiological changes to predict ATO before it occurred. Using ridge-regularized logistic regression (LR), we demonstrated that we could predict ATO 1 minute before it occurred using 3 minutes of preceding biosensor data with a mean area under the receiver operating characteristic curve (AUROC) of 0.71 for a population model (PM) and 0.84 for person-dependent models (PDMs). This study extends that initial research by replicating our approach in 70 independent participants with autism across 4 psychiatric inpatient units and expanding prediction evaluation to include self-injury and emotion dysregulation (ED; ie, tantrums and meltdowns).

## Methods

This prognostic study designed to update and validate a predictive model is reported following the Transparent Reporting of a Multivariable Prediction Model for Individual Prognosis or Diagnosis (TRIPOD) reporting guideline. The institutional review boards (IRBs) approved the Autism Inpatient Collection (AIC) and aggressive behavior prediction protocols of participating study sites (Bradley Hospital, Providence, Rhode Island; Cincinnati Children’s Hospital, Cincinnati, Ohio; Western Psychiatric Hospital, Pittsburgh, Pennsylvania; and Spring Harbor Hospital, Portland, Maine). IRB approval of the AIC extended to this study with an amendment. Guardians of all study participants provided informed consent.

### Study Participants

We enrolled 86 psychiatric inpatients not included in our previous studies serially at 4 clinical inpatient sites participating in the AIC (eMethods 1 in Supplement 1). Inclusion criteria included confirmation of autism via research-reliable administration of the Autism Diagnostic Observation Schedule-2 (ADOS-2)^26^ and parent-reported, staff-reported, or staff-observed physical aggression or self-injurious behavior (SIB). ADOS-2 is a semistructured autism diagnostic observation. In each of 4 developmental and language-level–dependent modules, a protocol of social presses is administered by a trained examiner, and then behavioral items relevant to autism spectrum disorders (ASDs) are scored as a standardized metric of severity ranging from 1 to 10 (with 1 indicating no ASD features and 10 indicating severe ASD symptoms). Exclusion criteria included not having a parent proficient in English or prisoner status for the individual with autism.

### Data Collection

Race and ethnicity were self-reported. Available race categories in the database were American Indian or Alaskan Native, Asian, Black or African American, Native Hawaiian or Other Pacific Islander, White, and other. Available ethnicity categories were Hispanic or Latino or not Hispanic or Latino. Race and ethnicity were assessed and included to describe sample characteristics. Intellectual disability was evaluated using the Leiter International Performance Scale–Third Edition (Leiter-3), a widely used standardized (normative mean [SD] score, 100 [15]) measure of nonverbal intellectual functioning designed to assess attention, cognition, and memory. It is administered without vocal instructions and does not require reading, writing, or verbal responses. Naturalistic observational coding sessions were performed by research staff. At the same time, inpatient study participants with autism wore the commercially available and regulatory-compliant E4 biosensor (Empatica, Inc) on their nondominant wrist. The E4 records changes in peripheral autonomic (blood volume pulse and electrodermal activity) and motion activity (3-axis acceleration) (eMethods 2 in Supplement 1). Research staff conducted observations with minimal interference in participant daily inpatient routines, which included academic lessons; behavioral, occupational, speech, and milieu therapies; meals; and free time. Research staff coded targeted aggressive behavior (ie, SIB, ED, and ATO) (see eMethods 3 in Supplement 1 for operational definitions) episode start (onset) and stop (offset) times within the observation period (eMethods 12 and eFigure 1 in Supplement 1) using a custom mobile application time-synchronized to the internal clock of the biosensor worn by participants.

### Statistical Analysis

After time-series feature extraction and data preprocessing (eMethods 4-5 in Supplement 1), we used ridge-regularized LR, support vector machines (SVMs), and neural networks (NNs) with extracted time-series features as input variables to make binary aggressive behavior predictions over time (eMethods 6-8 in Supplement 1). Specifically, at every time *t,* the classifier indicated the likelihood of aggressive behavior, indicated by the label *l*, in an upcoming time range (*t* to *t* + *τ_f_*), using features extracted in a previous time range (*t* − *τ_p_* to *t*). We also include the SD of each extracted feature in all prediction models. We use augmented feature vectors (AFVs) to refer to aggressive behavior observations and time since the last aggressive behavior, in contrast to features vectors (FVs), which do not include such labels. Data were analyzed from March 2020 through October 2023.

#### Classification Strategies

We performed aggressive behavior prediction using PMs and PDMs, wherein data were processed every 15 seconds for decision-making (eMethods 9 in Supplement 1). A single classifier was trained in PM using the entire data set, which included all participants and sessions. Individual classifiers were trained across sessions from a single participant in a PDM. In both models, 1 to 3 minutes of prior data (τ*_p_*) were used to make predictions 1 to 3 minutes into the future (τ*_f_*) given that this temporal range accommodated the briefest individual observational coding session in our corpus.

To achieve PM individualization, we explored the application of pseudolabeling as a domain adaptation (DA) technique comprising 2 phases: pseudolabeling and model training (eMethods 10 in Supplement 1). By using this iterative process, we aimed to enhance the adaptability and accuracy of PMs, ultimately improving their individualization for specific people.

#### Model Validation

We used AUROC values as our primary model performance metrics in all experiments. ROC curves plot the probability of false alarms vs the likelihood of detecting an event of interest at varying prediction thresholds. An ideal classifier presents a probability of detection equal to 1 for a probability of a false alarm equal to zero, and thus an AUROC of 1. For PDM models, we report mean AUROCs across all individuals and data splits. We computed ROCs and AUROCs for each discrete aggressive behavior in the multiclass setting. When evaluating DA, we calculated the median AUROC increase across participants.

#### Training and Testing Data-Splitting Methods

To avoid overfitting and to assess internal and external validity, we split the data 3 ways: session splits (SSs), *k*-fold cross-validation (CV) with leave-individuals-out (LIO), and CV with leave-sessions-out (LSO). Unless otherwise stated, we used 5-folds with 5 repetitions when using CV. We used SS in PM and PDM models, CV with LIO only in PM, and CV with LSO only in PDM. We split sessions in 2, using the first 80% of each session to construct the training set and reserving the remaining 20% for testing (eMethods 11 in Supplement 1). For experiments with CV, we computed 95% CIs for AUROCs obtained for all CV splits.

#### Experiments

We conducted 7 main experiments to evaluate AFVs and FVs (eMethods 12 in Supplement 1). Except for experiments 5 and 6, all investigations were performed with target aggressive behaviors combined into a single label (ie, CMB). The additional eighth experiment evaluated semisupervised DA for model individualization for different τ*_p_* and τ*_f_* settings with AFVs and FVs (eMethods 13 in Supplement 1). This post hoc analysis examines changes in AUROC as a function of observation duration and mean aggressive behavior frequency, duration, and intensity (eMethods 14 in Supplement 1).

## Results

### Data Collected

Of 86 participants enrolled, 16 individuals were not included in data analysis (18.6%) because they could not wear the physiological biosensor (8 individuals) or were discharged before an observation could be made (8 individuals). Common reasons stated by clinical staff for participants not being able to wear the sensor were tactile sensitivity and general behavioral noncompliance. There were 70 remaining study participants (mean [range; SD] age, 11.9 [5-19; 3.5] years; 62 males [88.6%]; 1 Asian [1.4%], 5 Black [7.1%], 1 Native Hawaiian or Other Pacific Islander [1.4%], and 63 White [90.0%]; 5 Hispanic [7.5%] and 62 non-Hispanic [92.5%] among 67 individuals with ethnicity data) (Table 1; see eMethods 15 in Supplement 1 for additional participant demographics). Our study population sex demographics were commensurate with the sex distribution of autism, wherein males are 4 times as likely as females to receive a diagnosis.^1^ Nearly half of the population (32 individuals [45.7%]) was minimally verbal (ADOS-2 module 1 or 2), and 30 individuals (42.8%) had an intellectual disability (mean [SD] Leiter-3 global IQ score, 72.96 [26.12]). Participant length of inpatient hospital stay ranged from 8 to 201 days (mean [SD] length of stay, 37.28 [33.95] days).

**Table 1.** Sample Characteristics

Characteristic	Participants, No. (%) (N = 70)
Age, mean (SD), y	11.9 (3.5)
Sex	
Male	62 (88.6)
Female	8 (11.4)
Race	
American Indian or Alaskan Native	0
Asian	1 (1.4)
Black or African American	5 (7.1)
Native Hawaiian or Other Pacific Islander	1 (1.4)
White	63 (90.0)
Other	0
Ethnicity	
Total with data, No.	67
Hispanic or Latino	5 (7.5)
Not Hispanic or Latino	62 (92.5)
ADOS-2 module^a^	
Module 1	25 (35.7)
Module 2	7 (10.0)
Module 3	32 (45.7)
Module 4	6 (8.6)
Leiter-3 nonverbal global IQ^b^	
Mean (SD)	72.96 (26.12)
120-139	2 (2.9)
110-119	3 (4.3)
90-109	14 (20.0)
80-89	7 (10.0)
70-79	5 (7.1)
60-69	9 (12.9)
50-59	5 (7.1)
40-49	8 (11.4)
30-39	8 (11.4)
Missing	9 (12.9)
Length of stay	37.28 (33.95)
Mean (SD), d	37.28 (33.95)
Missing, No.	1
Child Behavior Checklist–*DSM*–oriented scale, mean (SD), *t* score^c^	
Depressive problems	69.4 (9.5)
Oppositional defiant problems	68.2 (8.3)
Conduct problems	67.6 (9.2)
Attention-deficit/hyperactivity disorder problems	68.2 (7.6)
Anxiety problems	67.4 (10.4)
Somatic problems	60.8 (9.9)
Missing, No.	22
Vineland 3 Adaptive Behavior Scales, mean (SD)^d^	
Standard score	
Daily living skills	57.5 (19.4)
Communication	56.8 (23.6)
Socialization	46.5 (18.2)
*v* Scale score	
Internalizing	21.0 (1.4)
Externalizing	21.2 (1.8)
Missing, No.	24
Aberrant Behavior Checklist at admission, mean (SD)^e^	
Irritability	28.0 (9.6)
Stereotypy	7.7 (5.1)
Lethargy	14.7 (8.9)
Hyperactivity	28.5 (11.7)
Inappropriate speech	5.1 (3.3)
Missing, No.	12
Emotion dysregulation inventory at admission, mean (SD), *t* score^f^	
Reactivity	58.3 (7.9)
Dysphoria	58.0 (8.9)
Missing, No.	20

Abbreviations: ADOS-2, Autism Diagnostic Observation Schedule-2; *DSM*, *Diagnostic and Statistical Manual of Mental Disorders*; Leiter-3, Leiter International Performance Scale–Third Edition.

^a^ Module 1 is for individuals who do not possess consistent verbal communication skills. Module 2 is for individuals who have few communication skills. Module 3 is for individuals who are verbally fluent and capable of playing with age-appropriate toys and games. Module 4 is for individuals who are verbally fluent and seem to play with toys appropriate for individuals older than their age. Each ADOS-2 module is scored as a standardized metric of severity ranging from 1 to 10 (with 1 indicating no autism spectrum disorder features and 10 indicating severe autism spectrum disorder symptoms).

^b^ The Leiter-3 is a standardized IQ battery scored with a normative mean of 100 and SD of 15.

^c^ The Child Behavior Checklist is a 113-item parent-report measure of children’s psychiatric and behavioral functioning. It provides composite scale *t* scores for internalizing and externalizing problems. Higher scores indicate more problems in these areas; *t* scores have a mean of 50 and an SD of 10.

^d^ The Vineland Adaptive Behavior Scales, Second Edition is a standardized measure of adaptive functioning for individuals of any age. A primary parent or caregiver with knowledge of the child’s everyday routines and skills was asked to complete the parent or caregiver rating form. The Adaptive Behavior Composite score combines results from communication, daily living skills, socialization, and motor skills domains to provide an overall score of the child’s functioning level. Lower scores indicate greater impairment in adaptive functioning; *v* scale scores have a mean of 15 and an SD of 3.

^e^ The Aberrant Behavior Checklist is an informant rating instrument empirically derived by principal component analysis. It contains 58 items that resolve onto 5 subscales. Subscales and respective number of items include irritability (15 items), lethargy and social withdrawal (16 items), stereotypic behavior (7 items), hyperactivity and behavioral noncompliance (16 items), and inappropriate speech (4 items).

^f^ The Emotion Dysregulation Inventory is an informant questionnaire that assesses poor emotion regulation and is validated for general community and clinical populations, as well as youths with autism spectrum disorders. Response options ask respondents to rate each item on a 5-point Likert scale from “not at all” to “very severe” for observed functioning over the past 7 days. It includes 2 scales: Reactivity, which captures rapidly escalating, intense, and poorly regulated negative emotion and is available as a 24-item form or 7-item short form, and Dysphoria, a 6-item measure of low positive affect, sadness, and general unease.

After a brief desensitization protocol that involved gradually increasing exposure to the biosensor, 70 participants tolerated wearing the E4, and we obtained usable data for all participants. We collected 429 independent naturalistic observational coding sessions (median [IQR], 5 [1-7] sessions/participant) totaling 497 hours (median [IQR] duration, 4.46 [1.26-10.96] h/session) wherein concurrent E4 data were collected between March 2019 and March 2020. A total of 6665 aggressive behaviors were observed and annotated across participants, comprising 3983 episodes of SIB (59.8%; median [IQR], 2 [0-23] episodes/participant; median [IQR] duration, 1.97 [0.72-4.97] seconds), 2063 episodes of ED (31.0%; median [IQR], 8 [0-27] episodes/participant; median [IQR] duration, 10.09 [4.47-24.54] seconds), and 619 episodes of ATO (9.3%; median [IQR], 1 [0-8] episodes/per participant; median [IQR] duration, 2.31 [1.07-4.83] seconds) (Table 2). Episodes of SIB were thus 1.93 and 6.43 times more frequent than episodes of ED and ATO, respectively.

**Table 2.** Participant-Level Descriptive Statistics

Measure	Participants (N = 70)							
	Sessions		SIB episodes		ED episodes		ATO episodes	
	No.^a^	Duration, h	No.	Duration, s	No.	Duration, s	No.	Duration, s
Minimum	1	0.08	0	0.03	0	0.13	0	0.03
Maximum	25	37.72	1318	544.31	371	2017.16	27	674.25
Median	5	4.46	2	1.97	8	10.09	1	2.31
IQR	1-7	1.26-10.96	0-23	0.72-4.97	0-27	4.47-24.54	0-8	1.07-4.83
Total	429	497	3983	20 907	2063	64 017	619	5967

Abbreviations: ATO, aggression toward others; ED, emotion dysregulation; SIB, self-injurious behavior.

^a^ The bottom row displays the total overall sessions.

Interrater reliability analyses were conducted on data randomly selected from 20% of the total corpus (SIB: 796 episodes [20.0%]; ED: 412 episodes [20.0%]; ATO: 123 episodes [20.0%]) for each targeted aggressive behavior double-coded independently by 2 research staff within each inpatient site. These analyses yielded high agreement for SIB (κ = 0.93), ED (κ = 0.95), and ATO (κ = 0.86).

### Experiment Outcomes

**Figure 1.**
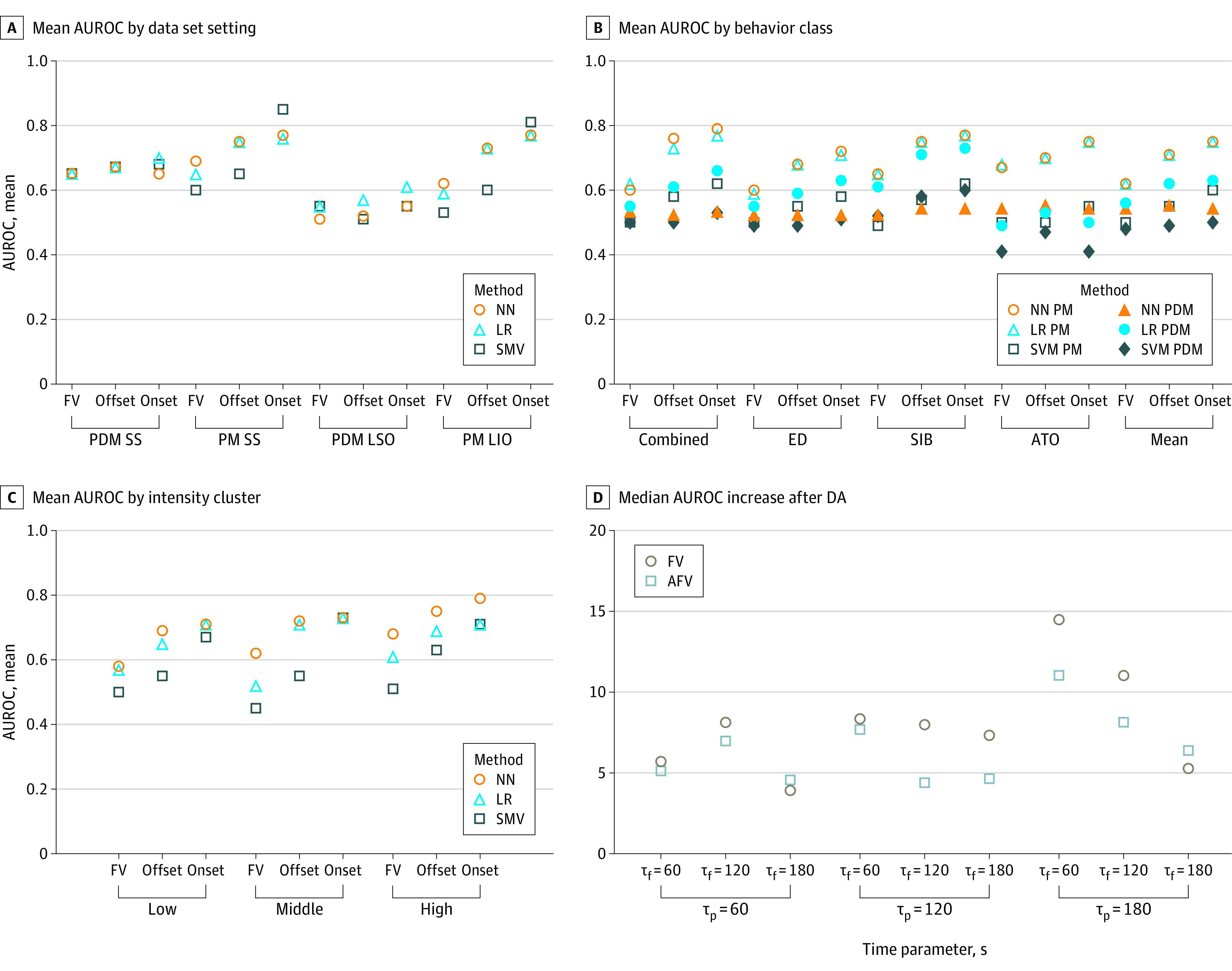
Mean Area Under the Receiver Operating Characteristic Curves (AUROCs) Across Time Parameter Values A, B, and C, Each marker represents the mean AUROC of 1 of 9 combinations of window lengths from the past (τ*_p_*) and in the future (τ*_f_*) (τ*_p_*, τ*_f_* ∈ {60,120,180} seconds). A, Mean AUROCs for experiments 1, 2, 3, and 4 for 3 scenarios (feature vectors [FVs], offset, and onset) are presented. B, Mean AUROC values of multiclass population models (PMs) with leave-individuals-out (LIO) (experiment 5) and multiclass person-dependent models (PDMs) with leave-sessions-out (LIO) (experiment 6) are presented. C, Mean AUROCs by aggressive behavior motion intensity cluster (experiment 7) are presented. D, Median AUROC changes after domain adaptation (DA) across data split settings are presented. Comparisons are made between PM performance on individual participant data before DA. AFV indicates augmented FV; ATO, aggression toward others; ED, emotion dysregulation; LR, logistic regression; NN, neural network; SIB, self-injurious behavior; SS, session split; SVM, support vector machine.

**Figure 2.**
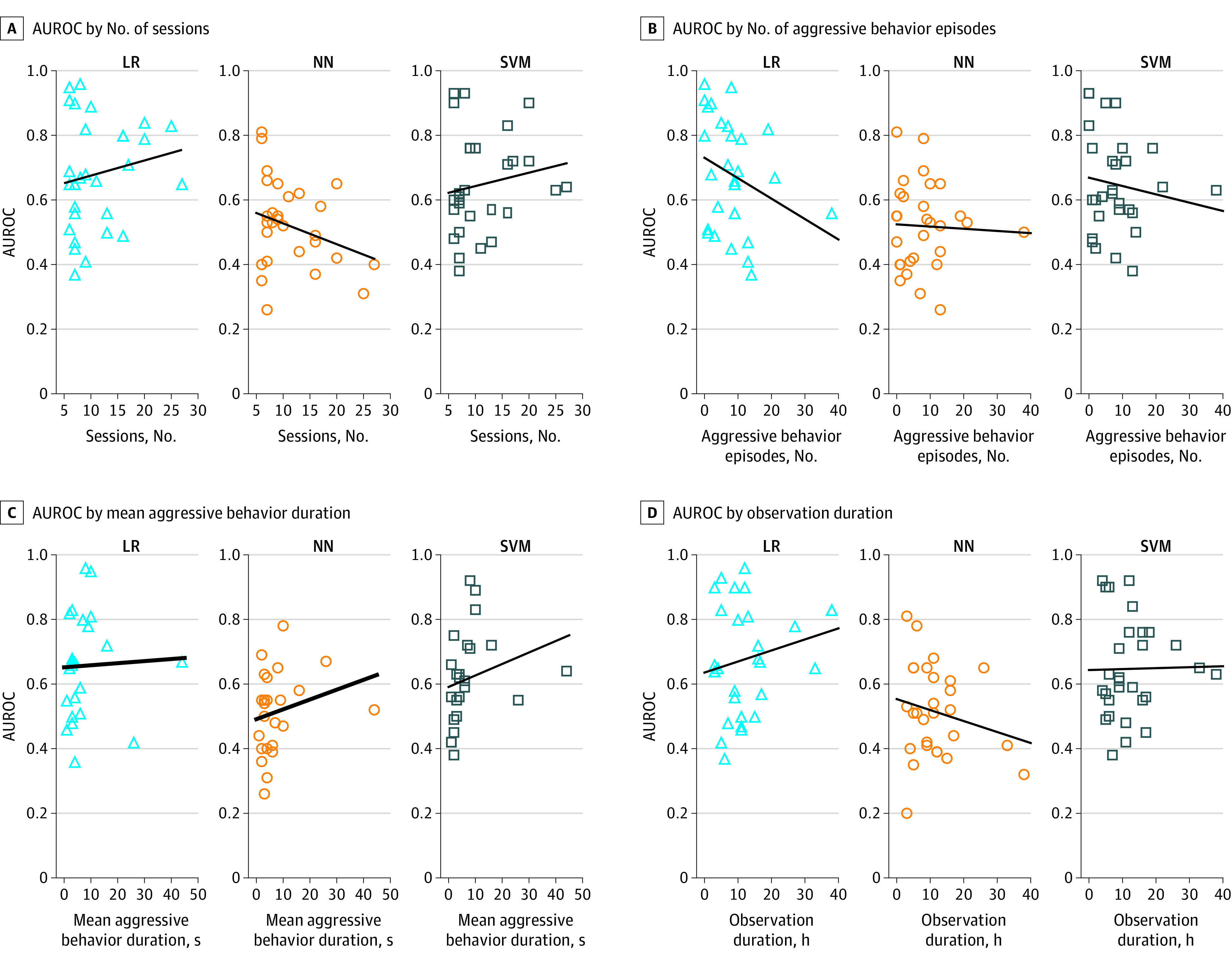
Aggressive Behavior Properties and Area Under the Receiver Operating Characteristic Curves (AUROCs) A, The AUROC by the number of participant observational sessions is presented. B, The AUROC by the number of aggressive behavior episodes is presented. C, The AUROC by the mean aggressive behavior episode duration is presented. D, The AUROC by the observation duration is presented. LR indicates logistic regression; NN, neural network; SMV, support vector machine.

The 8 experiments were performed with different values of τ*_p_* and τ*_f_*. Results are summarized in Figure 1 and Figure 2 and eResults 1, eFigure 1, and eFigure 2 in Supplement 1. Figure 1A to D presents mean AUROCs across all tested values of τ*_p_* and τ*_f_* (∈{60, 120, 180} seconds). We observed considerable performance improvements when using AFVs for all experiments, especially in the onset scenario.

Within the combined or binary classification setting (experiments 1-4), PM with SSs (experiment 1) produced the best results, followed by PM with LIO (experiment 3), using AFVs-onset. The best-performing classifier was SVM for PM with SS, achieving an AUROC of 0.87 (95% CI, 0.85-0.89). LR (AUROC = 0.81; 95% CI, 0.80-0.82) and NN (AUROC = 0.81; 95% CI, 0.79-0.83) reached the next best performance. For the longest prediction time in the future (τ*_f_* = 180 seconds), the best performance was achieved by SVM (AUROC = 0.85; 95% CI, 0.84-0.86), followed by LR (AUROC = 0.80; 95% CI, 0.79-0.81) and NN (AUROC = 0.78; 95% CI, 0.77-0.79), for PM with SS, and SVM (AUROC = 0.82; 95% CI, 0.77-0.87), followed by LR (AUROC = 0.77; 95% CI, 0.73-0.81) and NN (AUROC = 0.77; 95% CI, 0.73-0.81), for PM with LIO, both using AFVs-onset. LR and NN had comparable performance across PM experiments. They were often better than SVM for scenarios with FVs and AFV-offset. The performance of PM with LIO was similar to that of PM with SS. For instance, for a τ*_p_* of 180 seconds, a τ*_f_* of 60 seconds, and AFVs-offset, we obtained similar AUROCs for PM with SS (LR: 0.76; 95% CI, 0.75-0.77; NN: 0.76; 95% CI, 0.75-0.77; SVM: 0.66; 95% CI, 0.65-0.67) and PM with LIO (LR: 0.74; 95% CI, 0.68-0.80; NN: 0.75; 95% CI, 0.71-0.79; SVM: 0.60; 95% CI, 0.56-0.64). As τ*_f_* increased to 180 seconds (our largest prediction window), AUROCs oscillated in the models, with values of 0.75 (95% CI, 0.74-0.76) for LR, 0.75 (95% CI, 0.73-0.77) for NN, and 0.67 (95% CI, 0.66-0.68) for SVM for PM with SS and 0.73 (95% CI, 0.67-0.79) for LR, 0.73 (95% CI, 0.68-0.78) for NN, and 0.60 (95% CI, 0.53-0.67) for SVM for PM with LIO. We observed similar interactions for all τ*_p_* values. SS included 1 test set while LIO was performed with CV and thus may provide a more reliable estimation of test performance.

Figure 1A depicts the mean AUROC across all τ*_p_* and τ*_f_* for experiments 1 to 4. SVMs achieved superior performance for AFVs*-*onset. LR and NN performed similarly, with an advantage for LR in PDM with LSO. However, all methods performed poorly for PDM with LSO and better for PDM with SS. See eFigure 2 in Supplement 1 for AUROC curves for the best-performing NN, LR, and SVM for PM with SS and a τ*_f_* of 180 seconds. Estimating more exact compromises between true and false rates requires joint analysis with clinicians and caregivers and is beyond the scope of this study.

Experiments 5 and 6 leveraged PM with LIO and PDM with LSO, respectively, with a τ*_p_* of 180 seconds and considered multiclass SIB, ED, and ATO predictions. Results are summarized in Figure 1B. Compared with experiment 1 and 3 results, SVMs performed poorly even for AFVs-offset. Regarding PM with LIO, LR and NN models demonstrated similar performance, yielding the best results with AFVs-onset and a τ*_f_* of 60 seconds. We observed an LR AUROC value of 0.81 (95% CI, 0.78-0.84) for the combined class, indicating improvement compared with the binary scenarios of experiment 3. For AFVs-onset and a τ*_f_* of 180 seconds, we observed more comparable AUROCs for SIB (0.78; 95% CI, 0.70-0.86) and ATO (0.72; 95% CI, 0.64-0.80) than ED (0.69; 95% CI, 0.61-0.77). Among aggressive behaviors, SIB was most detectable with AFVs, while ATO was most detectable using FVs. For PDM with LSO (experiment 6) models, LR produced the best results, achieving a mean AUROC of 0.74 (95% CI, 0.73-0.75) for combined behaviors at a τ*_f_* of 180 seconds. Similar to what we found in experiment 2 and 4 results, we hypothesize that smaller data sets for NNs and SVMs may have been associated with lower AUROCs. Moving to individual classes, we observed higher AUROCs for SIB (0.69; 95% CI, 0.63-0.75) and ED (0.62; 95% CI, 0.55-0.69) than ATO (0.56; 95% CI, 0.52-0.60).

Experiment 7 evaluated the contribution of aggressive behavior motion intensity to prediction performance. Results obtained for a τ*_p_* of 180 seconds suggested that for mean outcomes (across τ*_p_* and τ*_f_* values), higher-intensity episodes were more detectable than low- and mid-intensity episodes, with NNs having higher AUROCs (Figure 1C; eMethods 14 in Supplement 1). For NN with AFVs-onset and a τ*_f_* of 60 seconds, AUROCs differed between low- and high-intensity scenarios (0.72; 95% CI, 0.65-0.79 vs 0.81; 95% CI, 0.75-0.87; difference = 12.5%) and mid- and high-intensity scenarios (0.75; 95% CI, 0.68-0.82 vs 0.81; 95% CI, 0.75- 0.87; difference = 8.0%).

Next, we evaluated AUROC variation associated with CMB properties: total observation duration, frequency, and aggressive behavior duration. Figure 2A-D illustrates results for PDM with SS using AFV-onset. Figure 2C illustrates positive correlations between mean aggressive behavior duration and AUROCs (LR: ρ = 0.26; NN: ρ = 0.22; SVM: ρ = 0.42). Figure 2B illustrates negative correlations between number of aggressive behavior episodes and AUROCs (LR: ρ = −0.40; NN: ρ = −0.07; SVM: ρ = −0.17). Figure 2A and D illustrate correlations of observation duration and number of sessions with AUROCs, where these quantities were positively correlated with AUROCs for LR (duration: ρ = 0.07; sessions: ρ = 0.12) and SVM (duration: ρ = 0.12; sessions: ρ = 0.19), while they were negatively correlated with the AUROCs for NN(duration: ρ = −0.10; sessions: ρ = −0.13).

We also evaluated LR in PM for the CMB scenario with CV on SS using AFV-onset test-retest reliability given that it was generally the best-performing model across experiments. We computed AUROC statistics (median and IQR) for a given session across individuals and CV splits, summarized in boxplots shown in eFigure 3 in Supplement 1. Given that the number of sessions varied by participant, we computed AUROCs by groups of 3 consecutive sessions, depicted in eFigure 3 in Supplement 1. Although a decreasing AUROC trend was apparent in both plots, grouping sessions was more stable than the median AUROC across session groups, ranging from 0.64 (0.62-0.72) for sessions 22 to 26 to 0.80 (0.77-0.82) for sessions 1 to 3.

Finally, we analyzed the use of semisupervised DA to mitigate the lack of data available for PDMs. In this experiment, we focused on LR owing to its overall reliability and considered FVs and AFVs-onset. Figure 1D presents median AUROC differences from the final individualized LR model after DA and the initial PM AUROC across varying training and test splits (eResults 2 and eFigure 4 in Supplement 1) at different τ*_p_* and τ*_f_* values. Across scenarios, we observed a noticeable increase in AUROC for the general PM. The best performance was achieved for a τ*_p_* of 180 seconds and τ*_f_* of 60 seconds, with median (IQR) AUROC improvements of 14.48 (11.37-17.08) and 11.03 (8.60-16.92) for FVs and AFVs-onset, respectively. The median (IQR) AUROC improvement for a τ*_p_* of 180 seconds and τ*_f_* of 180 seconds was 5.27 (2.05-7.18 ) and 6.38 (2.46-9.10) for FVs and AFVs-onset, respectively. For models with a τ*_f_* of 180 seconds, the maximum median (IQR) improvement in AUROC of 7.32 (5.13-9.85) was observed when τ*_p_* was 120 seconds for FVs. We highlight that these AUROC improvements were computed with respect to the initial PM model performance on individual data. As discussed in experiment 1, PMs with LIO and AFVs-onset had higher overall performance.

## Discussion

Our experiments in this prognostic study found that machine learning combined with wearable biosensing and time-stamped mobile behavior annotation data could be used to predict SIB, ED, and ATO in a sizable sample of youths with autism who were psychiatric inpatients. Our determination of best classifier performance considers the pattern of results observed across all experiments. While SVM produced the best single AUROC result, it did not perform as consistently well across all experiments as LR. Hence, we considered LR our best performing classifier. LR predictions yielded a mean 0.80 AUROC 3 minutes before aggressive behavior onset. Furthermore, our experiments demonstrated generalizability and reliability when we assessed model performance using LIO, LSO, and test-retest reliability approaches.

We also observed that knowledge regarding recent aggressive behavior was associated with improved prediction performance. Additionally, our results illustrated the ability to discriminate between different types and intensities of aggressive behavior; however, collapsing these 3 behaviors into 1 class was associated with better detection performance and lower false-positive rates. Of 3 aggressive behaviors evaluated, SIB was the most predictable. However, this may be a power issue given that SIB episodes were 1.93 and 6.43 times more frequent in our sample than ED and ATO episodes, respectively.

We also noticed a general decrease in performance when we extracted augmented features based on aggressive behavior offsets and removed feature vectors that fell within an aggressive behavior period, suggesting that ground truth behavior labels and reinforcement learning may be promising areas to explore further in this domain. Using biosensor acceleration as a proxy for intensity, we found that mean high-intensity aggressive behaviors were more predictable than mean mid- and low-intensity episodes. Finally, our results suggest that domain adaptation may be associated with improved individualized PM prediction performance.

### Limitations

Our study has several limitations, including restricted participant demography and geography and nonuniform frequency and duration of observed aggressive behavior across participants. More extensive trials with a more diverse participant population, including individuals in the outpatient setting, will be required to evaluate the generalizability of our results and to establish further the relative association of the amount of training data with prediction performance.

In future work, we will explore more advanced machine learning methods to improve prediction performance. Although NNs and SVMs are popular alternatives to linear models, our practical experience with these classifiers suggests high probabilities of overfitting or results that do not outperform simpler methods, such as LR. However, a promising addition may be person-dependent, nonhomogeneous point-process priors that enable longer prediction windows into the future. Modeling different physiological response profiles and estimating their differential likelihood of future aggressive behavior using Markov models may also be a fruitful future direction.

## Conclusion

This prognostic study sought to define an ecologically valid approach for identifying objective indicators of impending aggressive behaviors in youths with autism who were psychiatric inpatients. Our results suggest that biosensor data and machine learning have the potential to redress an intractable problem for a sizable segment of the autism population who are understudied and underserved.^27,28,29^ Our findings may lay the groundwork for developing just-in-time adaptive intervention mobile health systems^30^ that may enable new opportunities for preemptive intervention. By focusing on reducing the unpredictability of aggressive behavior, we anticipate that this ongoing research program may enable inpatient youths with autism to more fully participate in their homes, schools, and communities.

## Notes

Supplement 1. **eMethods 1.** Autism Inpatient Collection **eMethods 2.** Wearable Biosensor **eMethods 3.** Operational Definitions of Target Aggressive Behaviors **eMethods 4.** Time-Series Feature Extraction **eMethods 5.** Data Preprocessing **eMethods 6.** Logistic Regression Classifier Model **eMethods 7.** Support Vector Machine Classifier Model **eMethods 8.** Neural Network Classifier Model **eMethods 9.** Classification Strategies **eMethods 10.** Domain Adaptation of Population Models **eMethods 11.** Training-Testing Data-Splitting Methods **eMethods 12.** Experiments and Performance Assessments **eMethods 13.** Domain Adaptation Train and Test Methods **eFigure 1.** Aggressive Behavior Episode Timeline **eMethods 14.** Aggressive Behavior Intensity Analysis Methods **eMethods 15.** Additional Study Participant Demographics **eResults 1.** Classification Results for Experiments 1-7 **eFigure 2.** Best-Performing Classifiers for Population Model With Session Splits Using Augmented Feature Vectors-Onset for 180-s Times **eFigure 3.** Best Overall Performing Classifier Across Sessions **eResults 2.** Results for Experiment 8 (Domain Adaptation)
eReferences.

Supplement 2.
Data Sharing Statement
