# Supplementary material for: Wearable Biosensing to Predict Imminent Aggressive Behavior in Psychiatric Inpatient Youths With Autism
Source: JAMA Netw Open. 2023 Dec 21;6(12):e2348898. doi: 10.1001/jamanetworkopen.2023.48898 (PMC10739066; doi:10.1001/jamanetworkopen.2023.48898)
Supplement: Supplement 1. — eMethods 1. Autism Inpatient Collection eMethods 2. Wearable Biosensor eMethods 3. Operational Definitions of Target Aggressive Behaviors eMethods 4. Time-Series Feature Extraction eMethods 5. Data Preprocessing eMethods 6. Logistic Regression Classifier Model eMethods 7. Support Vector Machine Classifier Model eMethods 8. Neural Network Classifier Model eMethods 9. Classification Strategies eMethods 10. Domain Adaptation of Population Models eMethods 11. Training-Testing Data-Splitting Methods eMethods 12. Experiments and Performance Assessments eMethods 13. Domain Adaptation Train and Test Methods eFigure 1. Aggressive Behavior Episode Timeline eMethods 14. Aggressive Behavior Intensity Analysis Methods eMethods 15. Additional Study Participant Demographics eResults 1. Classification Results for Experiments 1-7 eFigure 2. Best-Performing Classifiers for Population Model With Session Splits Using Augmented Feature Vectors-Onset for 180-s Times eFigure 3. Best Overall Performing Classifier Across Sessions eResults 2. Results for Experiment 8 (Domain Adaptation) eReferences. [file jamanetwopen-e2348898-s001.pdf]

Supplemental Online Content

Imbiriba T, Demirkaya A, Singh A, Erdogmus D, Goodwin MS. Wearable biosensing to predict imminent aggressive behavior in psychiatric inpatient youths with autism. *JAMA Netw Open*. 2023;6(12):e2348898. doi:10.1001/jamanetworkopen.2023.48898

eMethods 1. Autism Inpatient Collection
eMethods 2. Wearable Biosensor
eMethods 3. Operational Definitions of Target Aggressive Behaviors
eMethods 4. Time-Series Feature Extraction
eMethods 5. Data Preprocessing
eMethods 6. Logistic Regression Classifier Model
eMethods 7. Support Vector Machine Classifier Model
eMethods 8. Neural Network Classifier Model
eMethods 9. Classification Strategies
eMethods 10. Domain Adaptation of Population Models
eMethods 11. Training-Testing Data-Splitting Methods
eMethods 12. Experiments and Performance Assessments
eMethods 13. Domain Adaptation Train and Test Methods
eFigure 1. Aggressive Behavior Episode Timeline
eMethods 14. Aggressive Behavior Intensity Analysis Methods
eMethods 15. Additional Study Participant Demographics
eResults 1. Classification Results for Experiments 1-7
eFigure 2. Best-Performing Classifiers for Population Model With Session Splits Using Augmented Feature Vectors-Onset for 180-s Times
eFigure 3. Best Overall Performing Classifier Across Sessions
eResults 2. Results for Experiment 8 (Domain Adaptation)
eReferences.

© 2023 Imbiriba T et al. *JAMA Network Open*.

This supplemental material has been provided by the authors to give readers additional information about their work.

© 2023 Imbiriba T et al. *JAMA Network Open*.

1. Autism Inpatient Collection

The Autism Inpatient Collection (AIC) is an ongoing six-site study of over 1,300 children, adolescents, and young adults admitted to specialized psychiatric inpatient units for persons with autism and other developmental disorders. The complete methods of the AIC were previously published [1]. Briefly, patients 4–20 years old with a score of ≥12 on the Social Communication Questionnaire (SCQ) [2] or high suspicion of autism from the inpatient clinical treatment team were eligible for enrollment. The AIC phenotypic data are available to external investigators through SFARI Base [3] and will soon include genetic sequencing data.

2. Wearable Biosensor

The E4 weighs 40g and is made of durable polyurethane and polycarbonate materials that make it water and shockproof. It uses photoplethysmography (PPG) [4] to record blood volume pulse (BVP) data at 64 Hz, enabling derivation of heart rate and heart rate variability (a measure of variation in the beat-to-beat (IBI) interval [5]). Differences in PPG due to skin color and external light intensity are dynamically compensated for by device firmware. Device firmware also includes motion artifact removal and IBI detection algorithms that automatically discard non-prototypical beats without smoothing IBI sequences. The E4 records electrodermal activity (EDA) at 4 Hz with a 0.01–100 micro siemens range, reflecting autonomic innervation of sweat glands and alterations in sympathetic nervous system arousal [6-7]. The E4 records motion-based activity up to 8 g at 32 Hz using a 3-axis accelerometer (ACCx, ACCy, and ACCz).

3. Operational Definitions of Target Aggressive Behaviors

Name of Behavior	Operational Definition	Examples
Aggression Toward Others (ATO)	● Behavior that may cause injury or harm to others.<br>● Forceful physical contact with another person [8]	Hitting, kicking, biting, scratching, grabbing, pulling, pushing, spitting, hair pulling, headbutting, slapping, grabbing, or throwing objects at people.
Self-Injurious Behavior (SIB)	● Behavior that may cause injury to self.<br>● Repetitive motor movements that result in injury to the person or have the potential to inflict injury [9]	Hitting self, biting self, scratching self, poking or gouging own eye, banging head on objects/surfaces, banging other body parts on objects/surfaces, skin picking, slapping self, pulling out own hair/teeth.
Emotional Dysregulation (ED)	● Perseverative agitation.<br>● Rapidly escalating, intense, or labile negative affect and difficulty calming down.	Screaming, crying, yelling, whining, loud/excessive moaning, grimacing, swearing, growling, threatening others, thrashing on the ground, suicidal/homicidal statements, stomping feet

© 2023 Imbiriba T et al. *JAMA Network Open*.

4. Time-Series Feature Extraction

As in our prior work [10], the following E4 time-series features for BVP, EDA, and three-axis acceleration (ACCx, ACCy, ACCz) were extracted using successive 15-sec sliding windows: first, last, maximum, minimum, mean, and median value; the number of unique values; and sum, standard deviation, and variance of values falling in a window. Thus, for every 15-second bin, we created a time-dependent feature vector and a label indicating if one of the target aggressive behaviors (i.e., SIB, ED, ATO) occurred. We also extracted the following labels provided by research staff coding: aggressive behavior observation (indicating when an aggressive behavior occurred) and time since the last aggressive behavior (indicating time elapsed since the last observation of an aggressive behavior). We extracted both features by considering aggressive behavior *onset* and *offset* (see eMethod**12** for more details). In our previous work, we only considered onsets. In the present study, we evaluate both.

5. Data Preprocessing

We performed z-score normalization by subtracting samples from the mean and dividing them by their standard deviation. Preliminary experiments with dimensionality reduction using Principal Component Analysis led to inferior accuracies compared to z-score normalization only and, thus, are not reported.

6. Logistic Regression Classifier Model

At each fold, the Logistic Regression (LR) classifier is trained via maximum likelihood estimation for optimal ridge-regularized regression weights $\beta = \left[\beta_0, \beta_1, \dots, \beta_d\right]^T$, where d is the number of features. For prediction, the classifier generates probabilities for two classes $l = +1$ (aggressive behavior) and $l = -1$ (non-aggressive behavior) in the form:

$$P(x, \beta) = \frac{1}{1 + e^{-\beta^T x}}$$

where $x = [1, x_1, \dots, x_d]^T$ and corresponds to the concatenated feature vector from $\left(t - \tau_p, t\right)$.

7. Support Vector Machine Classifier Model

In each experiment and at each fold, Support Vector Machines (SVMs) are trained and tested using the same concatenated feature vectors x as LR. Thus, given a training set $D = \{(x_1, l_1), \dots, (x_N, l_N)\}$, the SVM is trained using LibSVM and Gaussian kernels. Probabilistic estimates are obtained by using a sigmoid function whose parameters were obtained by maximum likelihood [11]. Finally, defining the hypothesis H_0 and H_1 as being "non-aggression" and "aggression," respectively, the detector becomes:

$$p(l = 1|h(x)) \; = \frac{p(h(x)|l = 1)p(l = 1)}{\Sigma_{j=\pm 1} p(h(x)|l = j)p(l = j)} \lessgtr \xi$$

where $h(x) = \sum_{j \in S} l_j \alpha_j \kappa(x_j, x)$ corresponds to the SVM scoring function, $\alpha_j, j = 1, \dots, N,$ are the Lagrange multipliers introduced in the dual formulation of the SVMs [12-13], and $\xi > 0$ is the decision threshold.

8. Neural Network Classifier Model

Neural networks (NN) used consisted of multilayer perceptrons with three hidden layers. We used ReLu activation functions for the hidden layers and *softmax* for the output layer, thus, providing class-posterior probabilities $p(l = j|x), j \in \{-1,1\}$. NNs were trained and tested using the same data preprocessing and splitting methods used in SVMs and LRs. We trained the NN using the Adam algorithm, cross-entropy loss function, and Keras framework. We utilize dropout layers to mitigate overfitting of the training data.

9. Classification Strategies

We use binary *one vs. rest* [14] classification for multiclass prediction across all population model (PM) experiments. For person-dependent models (PDM), we only select individuals with at least six sessions due to a lack of data for some participants. We evaluate the classification of aggressive behavior in our models in two ways. First, we merge SIB, ED, and ATO into a combined label (CMB) for binary classification. Second, we explore multiclass prediction of discrete SIB, ED, and ATO using multiple binary classifications.

© 2023 Imbiriba T et al. *JAMA Network Open*.

10. Domain Adaptation of Population Models

For the Domain Adaptation (DA) experiments, we only included participants with six or more sessions wherein at least one or more aggression episodes occurred. Our rationale is to ensure that enough sessions per participant are available for model adaptation and to mitigate class imbalance. We randomly select and compute average metrics over five experiments per participant to estimate test performance and reduce bias due to session order. We also consider session data without labels (i.e., unsupervised) and perform multiple splits where we separate sessions to compose training and testing data. Thus, given a population dataset $D_0 = \{(x_1, l_1), \ldots, (x_N, l_N)\}$, where $l_i \in \{-1,1\}$ indicates presence/absence of an aggressive behavior and unlabeled person data $U = \{x_1, \ldots, x_{N_U}\}$ with N_U data points. We perform experiments for each participant using different data splits for training and testing to assess the influence the amount of data has on prediction performance, i.e., U into U_{train} and U_{test} disjoint sets by varying the number at every cross-validation (CV) in U_{train} from 1 to N-1, where N represents the total number of sessions in U. We also create five Monte Carlo realizations by randomizing sessions in U before data splitting. Then, we apply a semi-supervised DA approach based on pseudo-labeling the data using the following algorithm:

$\theta \rightarrow argmax(L(\theta, D_0))$	% train the population model
$D = D_0$	
Until all x in U_{train} is labeled do:	
$D_s = \{\}$	% initialize D_s as an empty set
For all $x \in U_{train}$:	% iterate overall x in U_{train}
$\hat{l} = argmax_l P(\hat{l}\|x; \theta)$	% generate pseudo-labels for all x in U_{train}
If $P(\hat{l}\|x; \theta) > \tau$:	% introduce threshold τ on the posterior
$D_s = D_s \cup \{x, \hat{l}\}$	% admit the pseudo label if the posterior > τ
$\theta \rightarrow argmax\ L(\theta, D = D_0 \cup D_s)$	% retrain the model with admitted pseudo labels

Using the above algorithm, we iteratively update the model with training set pseudo labels. We compute Area Under the Curve (AUC) using U_{test} and the corresponding labels. We compute average AUC across five random shuffles of U_{train}. We denote this as AUC performance of the DA algorithm for the respective split setting, i.e., the number of sessions used for DA. We measure the efficacy of the DA procedure by computing the difference between AUC obtained before (population initial model) and after DA. That is $AUC_{diff} = AUC_{DA} - AUC_{Pop}$, where AUC_{Pop} and AUC_{DA} are the mean AUC obtained with the initial population model and after DA, respectively. To summarize AUC difference across varying values of τ_p and τ_f using feature vectors (FVs) and augmented feature vectors (AFVs)-*Onset*, we compute the median of the AUC difference across all participants and at every split of the unlabeled data.

11. Training-Testing Data Splitting Methods

For PM, data splits are performed using CV with five folds and two repetitions. We randomly select different participants during each CV iteration to form the training and testing sets. We employ the results obtained from each CV split to calculate average metrics. We also conduct data splits for PDM using CV with five folds and two repetitions. Different sessions are randomly selected at every CV iteration to create training and testing sets. Once again, we compute average metrics using the results from each CV split. For this analysis, we only include participants with more sessions than the number of CV folds (i.e., five). The main motivation for the different data split paradigms used is twofold: (1) to ensure data for training and testing never overlap, thus avoiding model overfitting, and (2) to create scenarios where data from all sessions appear in the training splits in the PDM scenario, thus mimicking a calibration period within each session. In CV splits, leave-individuals out and leave-sessions-out prevent data overlap between training and testing splits. In 80/20 splits, we ensure that data from all sessions appear in the training (calibration) and test sets while guaranteeing that training and test data do not overlap.

12. Experiments and Performance Assessments

For experiments 1-5, we consider two scenarios wherein (1) AFVs are extracted using onsets of CMB and (2) AFVs are extracted using offsets of CMB, in addition to removing FVs during CMB. The purpose of the second scenario is to simulate a real-world environment where a human could provide *posterior* aggressive behavior occurrence information to the classifier. Furthermore, we remove FVs and AFVs extracted for times t falling *within* an ongoing aggressive behavior. In Experiments 1-4, we consider different window lengths in the past ($\tau_p \in [60, 120, 180]$

© 2023 Imbiriba T et al. *JAMA Network Open*.

seconds) and different window lengths in the future ($\tau_f \in [60, 120, 180]$ seconds) for both onset/offset aggressive behavior scenarios. In Experiments 5, 6, and 7, we fix $\tau_p = 180$ and vary τ_f by 60, 120, 180 seconds. **eFigure1** visually represents a participant's aggressive behavior (AB) episode timeline, illustrating how we calculate behavioral onsets and offsets.

eFigure1. Aggressive Behavior Episode Timeline

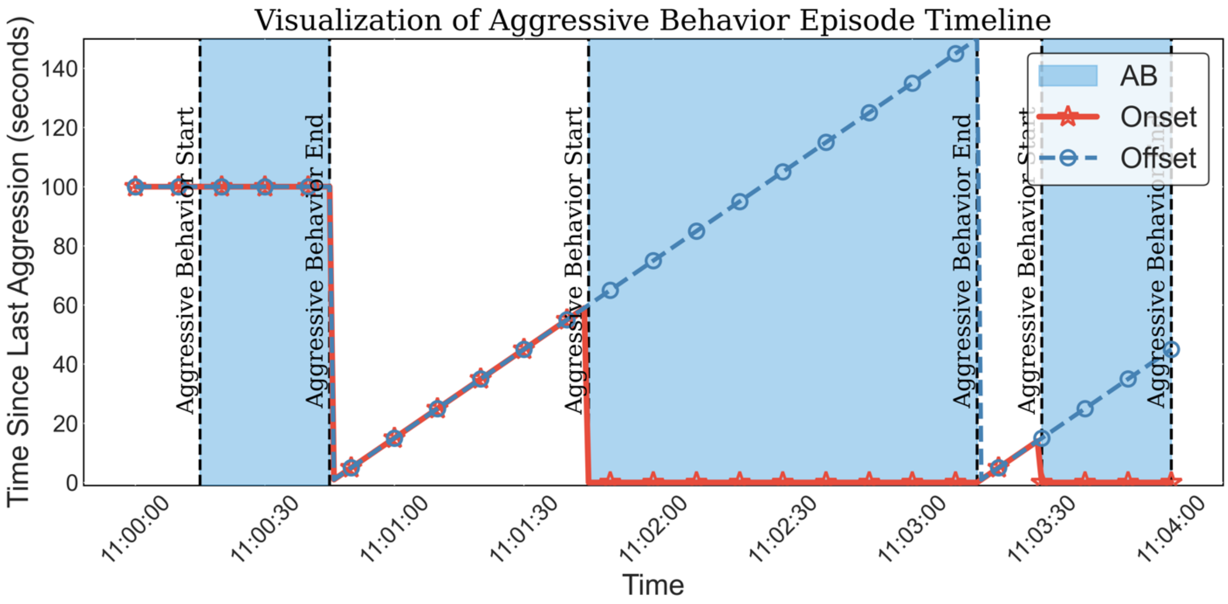

13. Domain Adaptation Train and Test Methods

We perform DA with a different number of train and test sessions from 1 to N-1, where N is the total number of sessions for individual participants with seven or more sessions (N = 26). Train and test sets are randomly selected before each repetition to avoid session order bias. We use the median AUC difference between the final individualized and initial population models across different data splits to assess performance.

14. Aggressive Behavior Intensity Analysis Methods

To quantify the intensity of observed aggressive behavior episodes, we use the E4 accelerometer (ACC_x, ACC_y, ACC_z) time-series to build a new feature time-series $NACC_t = \left(ACC_{x,t}^2 + ACC_{y,t}^2 + ACC_{z,t}^2\right)^{1/2}$ that quantifies the square root of the energy of XYZ movements over time. This time-series was averaged for every $\tau =$ 15 second bin resulting in a unidimensional temporal feature $\gamma_k^{(NACC)}$, with $k \in Z^+$ being the time index such that $t = k\tau$. Cluster analysis on this new feature ($\gamma_k^{(NACC)}$) provides more detailed labels for observed aggression episodes, wherein three clusters sorted into *low*, *moderate*, and *high* intensities are derived for each participant using the K-Means algorithm [15].

15. Study Participants

At admission, a primary caregiver of a study participant completed the Aberrant Behavior Checklist (ABC) [16], Child Behavior Checklist (CBCL) [17], Vineland Adaptive Behavior Scales-2 (VABS-2) [18], and Emotion Dysregulation Inventory (EDI) [19]. The Leiter-3 test of nonverbal intelligence [20] was also administered. Mean scores on the CBCL were in the borderline clinical or clinical range across psychiatric diagnostic categories. Mean VABS-2 scores in daily living skills, communication, and socialization were in the severely impaired range. Average ABC subscale scores were elevated, indicating a high frequency and severity of aggressive behavior (ABC irritability subscale M = 28.0, SD = 9.6). Reactivity (t-score M = 58.3, SD = 7.9) and dysphoria (t-score M = 58.0, SD = 8.9) scores on the EDI were in the average range compared to the EDI's large autism psychometric sample.

16. eResults 1: Classification results for Experiments 1-7.

© 2023 Imbiriba T et al. *JAMA Network Open*.

We performed Experiments 1-7 considering three scenarios. In scenario 1, we only utilize feature vectors. In scenario 2, we construct augmented features based on offsets of aggressive behaviors and remove feature vectors that end within positive episodes. In scenario 3, we construct augmented features (i.e., mobile behavior annotations) using onsets of aggressive behaviors and retain feature vectors that end within a positive episode. We highlight in bold the classifiers yielding the best performance (i.e., highest average AUC accuracy using the least amount of data from the past to make a prediction farthest into the future).

eTable 1. Classification results for Experiments 1-7.

**Ex1: Population Models with Session Splits (PM-SS), 80% training, 20% testing**										
		Feature Vectors			Augmented Feature Vectors (Offset)			Augmented Feature Vectors (Onset)		
		$\tau_f =$ 60	$\tau_f =$ 120	$\tau_f =$ 180	$\tau_f =$ 60	$\tau_f =$ 120	$\tau_f =$ 180	$\tau_f =$ 60	$\tau_f =$ 120	$\tau_f =$ 180
LR	$\tau_p = 60$	0.66	0.64	0.63	0.75	0.74	0.74	0.80	0.79	0.78
	$\tau_p = 120$	0.67	0.65	0.65	0.76	0.74	0.74	0.81	0.80	0.79
	$\tau_p = 180$	0.68	0.66	0.66	0.76	0.75	0.75	0.81	0.81	**0.80**
NN	$\tau_p = 60$	0.69	0.68	0.68	0.76	0.75	0.75	0.80	0.79	**0.78**
	$\tau_p = 120$	0.70	0.69	0.69	0.76	0.75	0.75	0.80	0.79	0.78
	$\tau_p = 180$	0.70	0.69	0.68	0.76	0.75	0.75	0.81	0.80	0.78
SVM	$\tau_p = 60$	0.54	0.59	0.52	0.63	0.64	0.64	0.87	0.86	**0.85**
	$\tau_p = 120$	0.56	0.55	0.56	0.65	0.65	0.66	0.86	0.84	0.83
	$\tau_p = 180$	0.56	0.56	0.43	0.66	0.66	0.67	0.87	0.83	0.81

© 2023 Imbiriba T et al. *JAMA Network Open*.

Ex2: Person-Dependent Models with Session Splits (PDM-SS)										
		Feature Vectors			Augmented Feature Vectors (Offset)			Augmented Feature Vectors (Onset)		
		$\tau_f =$ 60	$\tau_f =$ 120	$\tau_f =$ 180	$\tau_f =$ 60	$\tau_f =$ 120	$\tau_f =$ 180	$\tau_f =$ 60	$\tau_f =$ 120	$\tau_f =$ 180
LR	$\tau_p = 60$	0.67	0.68	0.65	0.71	0.70	0.67	0.74	0.72	**0.70**
	$\tau_p = 120$	0.67	0.65	0.64	0.69	0.67	0.65	0.73	0.70	0.68
	$\tau_p = 180$	0.65	0.64	0.64	0.67	0.65	0.65	0.70	0.67	0.67
NN	$\tau_p = 60$	0.66	0.65	0.66	0.67	0.69	0.66	0.56	0.67	**0.68**
	$\tau_p = 120$	0.67	0.58	0.64	0.66	0.67	0.65	0.68	0.68	0.68
	$\tau_p = 180$	0.67	0.67	0.66	0.67	0.69	0.68	0.70	0.67	0.55
SVM	$\tau_p = 60$	0.65	0.62	0.60	0.67	0.69	0.66	0.67	0.67	**0.68**
	$\tau_p = 120$	0.70	0.66	0.60	0.66	0.67	0.65	0.68	0.68	0.68
	$\tau_p = 180$	0.70	0.67	0.65	0.67	0.69	0.68	0.70	0.67	0.66

© 2023 Imbiriba T et al. *JAMA Network Open*.

Ex3: Population Models with Leave-Individuals-Out (PM-CV-LIO)										
		Feature Vectors			Augmented Feature Vectors (Offset)			Augmented Feature Vectors (Onset)		
		$\tau_f =$ 60	$\tau_f =$ 120	$\tau_f =$ 180	$\tau_f =$ 60	$\tau_f =$ 120	$\tau_f =$ 180	$\tau_f =$ 60	$\tau_f =$ 120	$\tau_f =$ 180
LR	$\tau_p = 60$	0.60 ±0.13	0.55 ±0.10	0.56 ±0.11	0.74 ±0.05	0.73 ±0.05	0.73 ±0.06	0.80 ±0.05	0.77 ±0.05	0.76 ±0.04
	$\tau_p = 120$	0.59 ±0.08	0.57 ±0.05	0.56 ±0.08	0.74 ±0.06	0.73 ±0.05	0.73 ±0.06	0.80 ±0.05	0.78 ±0.05	**0.77** ±0.04
	$\tau_p = 180$	0.60 ±0.08	0.58 ±0.08	0.58 ±0.08	0.74 ±0.06	0.73 ±0.06	0.73 ±0.05	0.80 ±0.05	0.80 ±0.04	0.76 ±0.06
NN	$\tau_p = 60$	0.61 ±0.05	0.60 ±0.04	0.59 ±0.06	0.72 ±0.03	0.71 ±0.05	0.71 ±0.04	0.78 ±0.04	0.77 ±0.04	0.76 ±0.04
	$\tau_p = 120$	0.62 ±0.03	0.62 ±0.03	0.60 ±0.04	0.74 ±0.04	0.73 ±0.04	0.71 ±0.04	0.78 ±0.04	0.77 ±0.04	**0.77** ±0.04
	$\tau_p = 180$	0.64 ±0.04	0.62 ±0.06	0.62 ±0.06	0.75 ±0.04	0.73 ±0.04	0.73 ±0.03	0.77 ±0.04	0.77 ±0.04	0.76 ±0.04
SVM	$\tau_p = 60$	0.53 ±0.05	0.53 ±0.05	0.56 ±0.06	0.58 ±0.03	0.58 ±0.03	0.59 ±0.04	0.83 ±0.05	0.82 ±0.05	0.81 ±0.05
	$\tau_p = 120$	0.52 ±0.05	0.54 ±0.05	0.56 ±0.05	0.59 ±0.03	0.59 ±0.04	0.59 ±0.04	0.81 ±0.05	0.80 ±0.05	**0.82** ±0.05
	$\tau_p = 180$	0.52 ±0.05	0.55 ±0.05	0.55 ±0.05	0.60 ±0.04	0.60 ±0.03	0.60 ±0.04	0.81 ±0.06	0.80 ±0.06	0.79 ±0.05

© 2023 Imbiriba T et al. *JAMA Network Open*.

Ex4: Person-Dependent Models with Leave-Sessions-Out (PDM-CV-LSO)										
		Feature Vectors			Augmented Feature Vectors (Offset)			Augmented Feature Vectors (Onset)		
		$\tau_f =$ 60	$\tau_f =$ 120	$\tau_f =$ 180	$\tau_f =$ 60	$\tau_f =$ 120	$\tau_f =$ 180	$\tau_f =$ 60	$\tau_f =$ 120	$\tau_f =$ 180
LR	$\tau_p = 60$	0.57 ±0.02	0.57 ±0.03	0.57 ±0.05	0.63 ±0.03	0.63 ±0.01	0.62 ±0.03	0.68 ±0.02	0.65 ±0.01	**0.65** ±0.03
	$\tau_p = 120$	0.59 ±0.03	0.59 ±0.04	0.57 ±0.04	0.64 ±0.03	0.64 ±0.02	0.62 ±0.01	0.68 ±0.02	0.66 ±0.02	0.64 ±0.04
	$\tau_p = 180$	0.60 ±0.03	0.59 ±0.03	0.58 ±0.03	0.64 ±0.03	0.63 ±0.03	0.62 ±0.05	0.68 ±0.03	0.65 ±0.03	0.64 ±0.03
NN	$\tau_p = 60$	0.51 ±0.01	0.51 ±0.02	0.51 ±0.03	0.52 ±0.02	0.51 ±0.02	0.52 ±0.03	0.56 ±0.02	0.55 ±0.03	**0.56** ±0.03
	$\tau_p = 120$	0.51 ±0.02	0.52 ±0.02	0.52 ±0.03	0.52 ±0.02	0.51 ±0.03	0.52 ±0.03	0.54 ±0.02	0.54 ±0.03	0.54 ±0.02
	$\tau_p = 180$	0.52 ±0.02	0.52 ±0.03	0.51 ±0.02	0.52 ±0.03	0.52 ±0.03	0.52 ±0.03	0.53 ±0.01	0.53 ±0.03	0.52 ±0.03
SVM	$\tau_p = 60$	0.55 ±0.03	0.55 ±0.04	0.54 ±0.04	0.51 ±0.04	0.51 ±0.04	0.51 ±0.04	0.55 ±0.04	0.55 ±0.04	**0.56** ±0.05
	$\tau_p = 120$	0.53 ±0.03	0.52 ±0.03	0.54 ±0.04	0.51 ±0.02	0.51 ±0.04	0.51 ±0.04	0.55 ±0.04	0.55 ±0.04	0.55 ±0.05
	$\tau_p = 180$	0.55 ±0.03	0.53 ±0.04	0.53 ±0.04	0.51 ±0.04	0.51 ±0.03	0.51 ±0.04	0.54 ±0.04	0.55 ±0.04	0.55 ±0.05

© 2023 Imbiriba T et al. *JAMA Network Open*.

Ex5: Multiclass Population Models with Leave-Individuals-Out (MC-PM-CV-LIO), TP = 180										
		Feature Vectors			Augmented Feature Vectors (Offset)			Augmented Feature Vectors (Onset)		
		$\tau_f =$ 60	$\tau_f =$ 120	$\tau_f =$ 180	$\tau_f =$ 60	$\tau_f =$ 120	$\tau_f =$ 180	$\tau_f =$ 60	$\tau_f =$ 120	$\tau_f =$ 180
LR	Combined	0.60	0.58	0.58	0.78	0.76	0.75	0.81	0.79	**0.79**
	ED	0.60	0.55	0.52	0.71	0.65	0.65	0.77	0.72	0.69
	SIB	0.54	0.53	0.53	0.78	0.77	0.75	0.80	0.78	**0.78**
	ATO	0.65	0.61	0.59	0.75	0.73	0.70	0.78	0.74	0.72
	Av. AUC	0.60	0.57	0.55	0.76	0.73	0.71	0.79	0.76	0.75
NN	Combined	0.64	0.62	0.62	0.75	0.74	0.73	0.79	0.78	0.77
	ED	0.62	0.56	0.56	0.71	0.65	0.66	0.77	0.71	0.69
	SIB	0.64	0.63	0.64	0.74	0.74	0.73	0.78	0.76	**0.77**
	ATO	0.69	0.66	0.67	0.74	0.70	0.68	0.77	0.76	0.71
	Av. AUC	0.65	0.62	0.62	0.73	0.71	0.70	0.78	0.75	0.73
SVM	Combined	0.50	0.50	0.50	0.59	0.58	0.57	0.64	0.63	0.62
	ED	0.49	0.50	0.51	0.56	0.55	0.54	0.59	0.58	0.57
	SIB	0.50	0.50	0.50	0.58	0.57	0.56	0.62	0.62	**0.62**
	ATO	0.50	0.50	0.50	0.56	0.55	0.54	0.56	0.55	0.54
	Av. AUC	0.50	0.50	0.50	0.57	0.56	0.55	0.61	0.60	0.59

© 2023 Imbiriba T et al. *JAMA Network Open*.

Ex6: Multiclass Person-Dependent Models with Leave-Sessions-Out (MC-PDM-CV-LSO), TP = 180										
		Feature Vectors			Augmented Feature Vectors (Offset)			Augmented Feature Vectors (Onset)		
		$\tau_f =$ 60	$\tau_f =$ 120	$\tau_f =$ 180	$\tau_f =$ 60	$\tau_f =$ 120	$\tau_f =$ 180	$\tau_f =$ 60	$\tau_f =$ 120	$\tau_f =$ 180
LR	Combined	0.58	0.57	0.57	0.64	0.64	0.63	0.69	0.68	0.66
	ED	0.59	0.57	0.54	0.64	0.60	0.59	0.70	0.65	0.62
	SIB	0.65	0.65	0.58	0.75	0.73	0.68	0.76	0.74	**0.69**
	ATO	0.46	0.47	0.56	0.51	0.51	0.58	0.47	0.48	0.56
	Av. AUC	0.55	0.58	0.63	0.63	0.64	0.73	0.64	0.65	0.74
NN	Combined	0.53	0.53	0.53	0.52	0.53	0.51	0.54	0.52	0.52
	ED	0.52	0.53	0.50	0.53	0.52	0.52	0.51	0.54	0.53
	SIB	0.50	0.54	0.53	0.55	0.54	0.52	0.52	0.55	0.53
	ATO	0.55	0.55	**0.57**	0.63	0.57	0.54	0.52	0.55	0.55
	Av. AUC	0.55	0.57	0.52	0.59	0.58	0.54	0.54	0.59	0.57
SVM	Combined	0.49	0.49	0.50	0.50	0.51	0.49	0.54	0.55	0.53
	ED	0.49	0.49	0.47	0.48	0.50	0.48	0.53	0.52	0.52
	SIB	0.53	0.52	0.53	0.57	0.57	0.59	0.59	0.60	**0.62**
	ATO	0.43	0.41	0.44	0.45	0.47	0.49	0.42	0.41	0.43
	Av. AUC	0.47	0.48	0.48	0.49	0.49	0.49	0.52	0.52	0.51

© 2023 Imbiriba T et al. *JAMA Network Open*.

**Ex7: Intensity Classification for Population Models with Behaviors Combined and Leave-Individuals Out (PM-CMB-CV-LIO), $\tau_p = 180$**										
		Feature Vectors			Augmented Feature Vectors (Offset)			Augmented Feature Vectors (Onset)		
		$\tau_f = 60$	$\tau_f = 120$	$\tau_f = 180$	$\tau_f = 60$	$\tau_f = 120$	$\tau_f = 180$	$\tau_f = 60$	$\tau_f = 120$	$\tau_f = 180$
LR	LOW	0.58	0.53	0.50	0.68	0.65	0.62	0.74	0.68	0.65
	MID	0.56	0.55	0.55	0.71	0.70	0.70	0.74	0.73	**0.73**
	HIGH	0.64	0.60	0.57	0.72	0.69	0.67	0.76	0.73	0.72
NN	LOW	0.60	0.57	0.61	0.70	0.67	0.66	0.72	0.72	0.72
	MID	0.63	0.64	0.62	0.74	0.74	0.72	0.75	0.77	0.72
	HIGH	0.66	0.64	0.67	0.74	0.73	0.71	0.81	0.73	**0.77**
SVM	LOW	0.50	0.49	0.50	0.55	0.55	0.54	0.68	0.67	**0.67**
	MID	0.50	0.46	0.47	0.54	0.55	0.53	0.74	0.73	0.72
	HIGH	0.53	0.52	0.51	0.64	0.63	0.63	0.73	0.72	0.71

© 2023 Imbiriba T et al. *JAMA Network Open*.

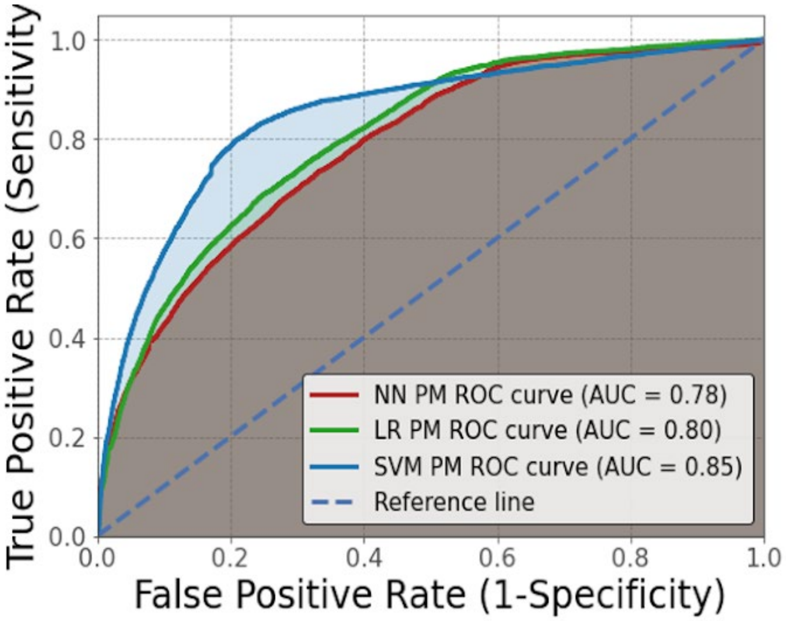

eFigure 2: ROC curves for the best-performing NN, LR, and SVM PM-SS-AFV-*Onset* for $\tau_p = \tau_f = 180$.

© 2023 Imbiriba T et al. *JAMA Network Open*.

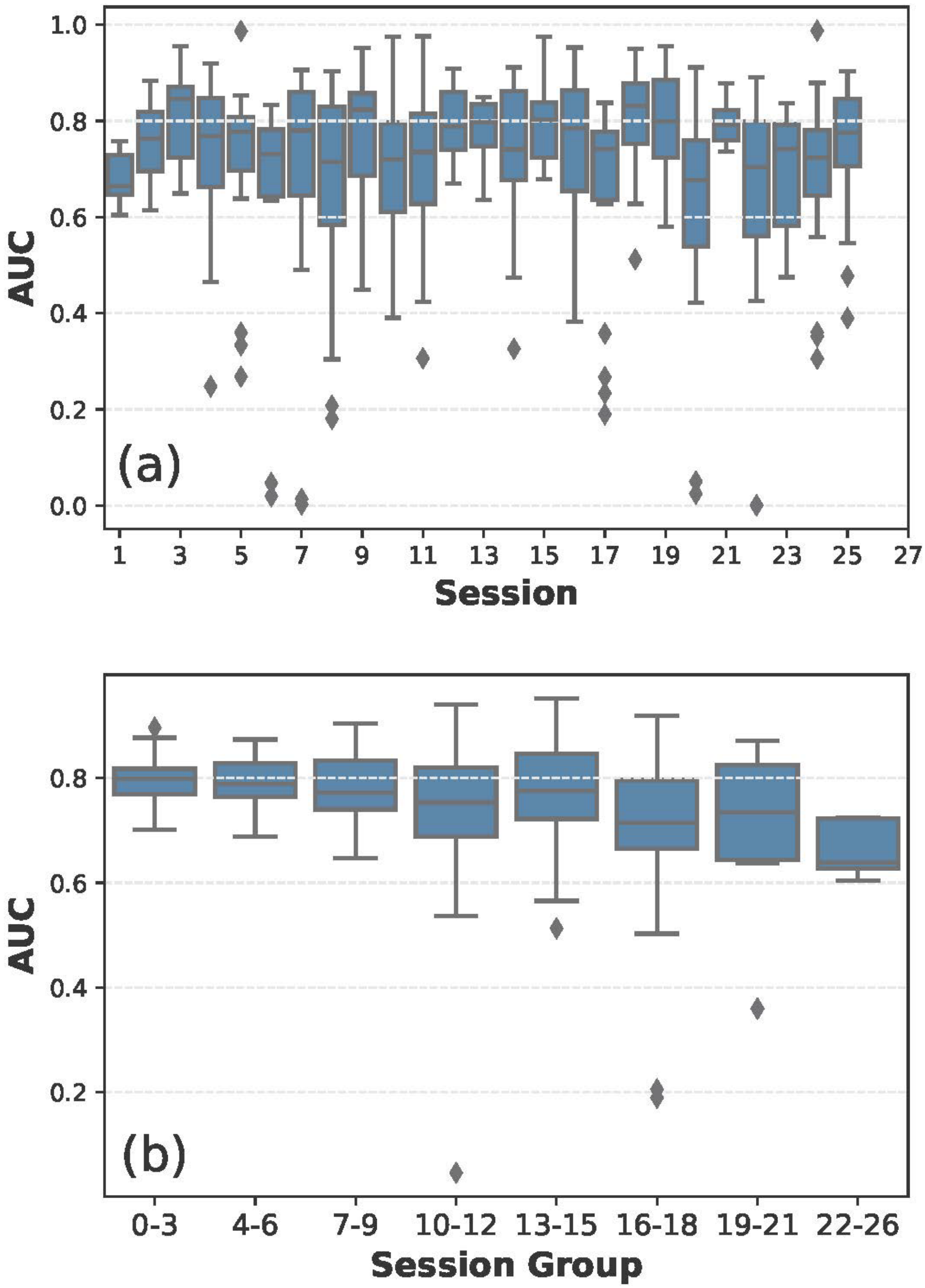

eFigure 3: (**a**) Performance of the best overall performing classifier, LR-PM-CV-LIO-AFV-*Onset* across sessions. (**b**) We computed the performance of LR-PM-CV-LIO-AFV-*Onset* by calculating AUC statistics over three subsequent sessions. We performed cross-validation for both experiments using 10 CV folds and 5 randomized repetitions.

© 2023 Imbiriba T et al. *JAMA Network Open*.

17. eResults2 for Experiment 8 (Domain Adaptation)

eFigure 4 presents the median AUC for DA across all individuals for different sessions splits, τ_p =180, τ_f = 60, AFV-*Onset* **(a)** and τ_p =180, τ_f = 180, AFV-*Onset* **(b)**. For predictions with a smaller future window length τ_f= 60 **(a)**, median AUC improvement is greater than the larger future window length τ_f = 180 **(b)**. In both cases, we observe a clear improvement in the median AUC when more data from the participant is used in the DA phase.

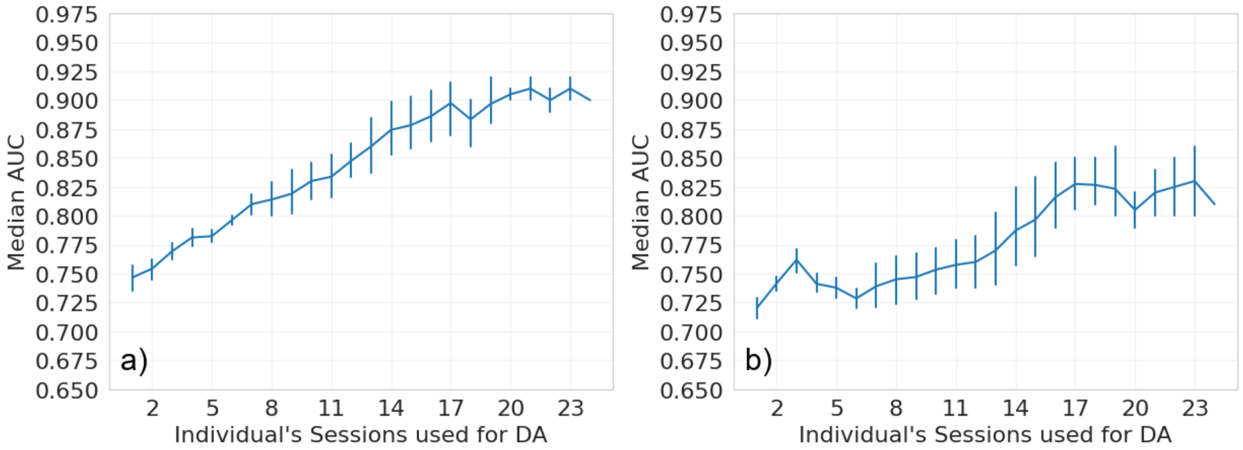

eFigure 4: Median (solid line) AUC of the model across all individuals after Domain Adaptation for τ_p =180, τ_f = 60, AFV-*Onset* **(a)** and τ_p =180, τ_f = 180, AFV-*Onset* **(b)**. The bars represent the IQR range of the median AUCs. Only one individual in the study had 24 sessions that could be used in the DA step; hence no confidence interval can be calculated.

© 2023 Imbiriba T et al. *JAMA Network Open*.

18. Supplement References

1. Siegel M, Smith KA, Mazefsky C, et al. The autism inpatient collection: methods and preliminary sample description. *Mol Autism*. 2015;6:61-71.

2. Rutter M, Bailey A, Lord C. *The social communication questionnaire: Manual*. Western Psychological Services; 2003.

3. SFARI Base. https://www.sfari.org/resource/sfari-base/. Accessed June 5, 2023.

4. Allen J. Photoplethysmography and its application in clinical physiological measurement. *Physiol Meas*. 2007;28(3):R1.

5. Electrophysiology, Task Force of the European Society of Cardiology the North American Society of Pacing. Heart rate variability: standards of measurement, physiological interpretation, and clinical use. *Circulation*. 1996;93(5):1043-1065.

6. Critchley HD. Electrodermal responses: what happens in the brain. *Neuroscientist*. 2002;8(2):132-142.

7. Boucsein W. *Electrodermal activity*. Springer Science & Business Media; 2012.

8. Mace FC, Prager KL, Thomas K, et al. Effects of stimulant medication under varied motivational operations. *J Appl Behav Anal*. 2009;42(1):177-183.

9. Lewis MH, Bodfish JW. Repetitive behavior disorders in autism. *Ment Retard Dev Disabil Res Rev*. 1998;4(2):80-89.

10. Goodwin MS, Mazefsky CA, Ioannidis S, Erdogmus D, Siegel M. Predicting aggression to others in youth with autism using a wearable biosensor. *Autism Res*. 2019;12(8):1286-1296.

11. Platt J. Probabilistic outputs for support vector machines and comparisons to regularized likelihood methods. *Advances in large margin classifiers.* 1999; 10(3), 61-74.

12. Suykens JA, De Brabanter J, Lukas L, Vandewalle, J. Weighted least squares support vector machines: robustness and sparse approximation. *Neurocomputing*. 2002; 48(1-4), 85-105.

13. Steinwart I, Christmann A. *Support vector machines*. Springer Science & Business Media. 2008.

14. Rifkin R, Klautau A. In defense of one-vs-all classification. *J Mach Learn Res.* 2004;5:101-141.

15. Arthur D, Vassilvitskii S. k-means++: The Advantages of Careful Seeding. Technical Report. Stanford; 2006.

16. Aman MG, Singh NN, Stewart AW, Field CJ. The aberrant behavior checklist: A behavior rating scale for the assessment of treatment effects. *Am J Ment Defic*. 1985;89(5):485-491.

17. Achenbach TM. *Manual for the Teacher's Report Form and 1991 profile*. University of Vermont Department of Psychiatry; 1991.

18. Sparrow SS, Balla DA, Cicchetti DV. *Vineland Adaptive Behavior Scales*. Circle Pines, MN: American Guidance Service; 1984.

19. Mazefsky CA, Yu L, White SW, Siegel M, Pilkonis PA. The emotion dysregulation inventory: Psychometric properties and item response theory calibration in an autism spectrum disorder sample. *Autism Res*. 2018;11(6):928-941.

© 2023 Imbiriba T et al. *JAMA Network Open*.

20. Roid GH, Koch C. Leiter-3: Nonverbal cognitive and neuropsychological assessment. In: Bracken BA, ed. *Handbook of Nonverbal Assessment*. Springer; 2017:127-150.

© 2023 Imbiriba T et al. *JAMA Network Open*.
