# Supplementary material for: Wearable Biosensing to Predict Imminent Aggressive Behavior in Psychiatric Inpatient Youths With Autism
Source: JAMA Netw Open. 2023 Dec 21;6(12):e2348898. doi: 10.1001/jamanetworkopen.2023.48898 (PMC10739066; doi:10.1001/jamanetworkopen.2023.48898)
Supplement: Supplement 2. — Data Sharing Statement [file jamanetwopen-e2348898-s002.pdf]

Data Sharing Statement

Imbiriba. Wearable Biosensing to Predict Imminent Aggressive Behavior in Psychiatric Inpatient Youths With Autism. *JAMA Netw Open*. Published online December 21, 2023. doi:10.1001/jamanetworkopen.2023.48898

Data
Data available: Yes
Data types: Deidentified participant data, Data dictionary
How to access data: Will be made available pre-production.
When available: With publication

Supporting Documents
Document types: Other (please specify)
Additional Information: Data access instructions, deidentified data description, data dictionary description, acceptable use policy
How to access documents: Will be made available pre-production.
When available: With publication

Additional Information
Who can access the data: Researchers whose proposed use of the data has been approved. **Types of analyses:** The scientific community for non-commercial research purposes **Mechanisms of data availability:** Per the Simons Foundation data sharing policy
